# Pharmacological Ascorbate Suppresses Growth of Gastric Cancer Cells with GLUT1 Overexpression and Enhances the Efficacy of Oxaliplatin Through Redox Modulation: Erratum

**DOI:** 10.7150/thno.114259

**Published:** 2025-04-19

**Authors:** Yun-Xin Lu, Qi-Nian Wu, Dong-liang Chen, Le-Zong Chen, Zi-Xian Wang, Chao Ren, Hai-yu Mo, Ya Chen, Hui Sheng, Ying-Nan Wang, Yun Wang, Jia-Huan Lu, De-shen Wang, Zhao-lei Zeng, Feng Wang, Feng-Hua Wang, Yu-Hong Li, Huai-Qiang Ju, Rui-Hua Xu

**Affiliations:** 1 Sun Yat-sen University Cancer Center, State Key Laboratory of Oncology in South China, Collaborative Innovation Center for Cancer Medicine, Guangzhou, 510060, China;; 2 Department of Medical Oncology, Sun Yat-sen University Cancer Center, Guangzhou, 510060, China;

The authors regret that incorrect flow images of MGC803 cells in Figure 1A and immunohistochemistry images of Ki-67 in Vit. and CPT11 group in Figure S7F was used during data arranging processes. The corrected Figures are shown below.

## Figures and Tables

**Figure A:**
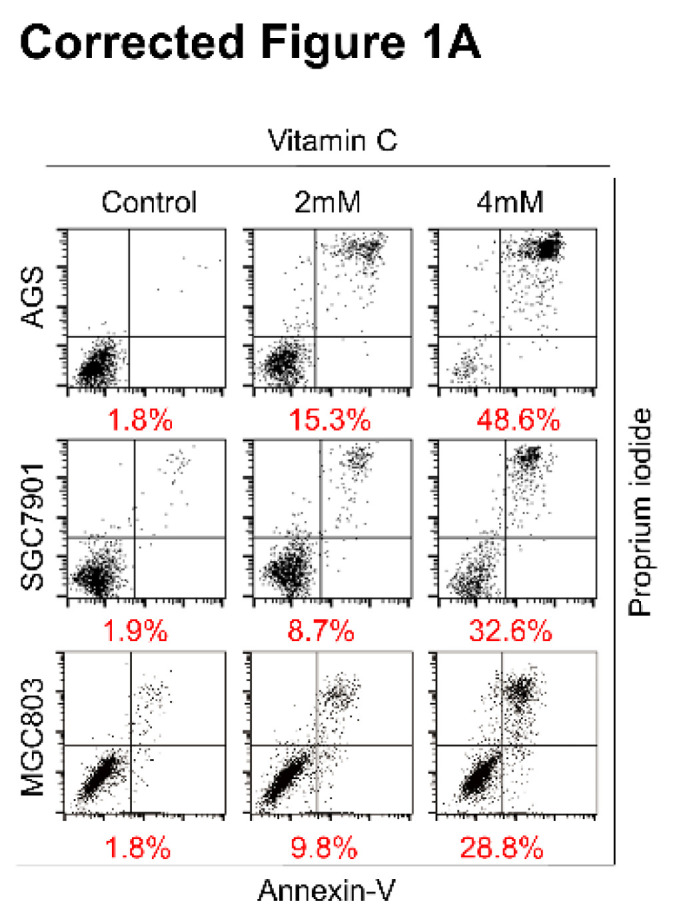
**Corrected Figure 1A** Ascorbate induces apoptosis and inhibits proliferation of gastric cancer cells. (A) Representative images of cell apoptosis in the indicated cells treated with ascorbate (Vitamin C, 2h) were determined by Annexin V/propidium iodide (PI) assays.

**Figure B:**
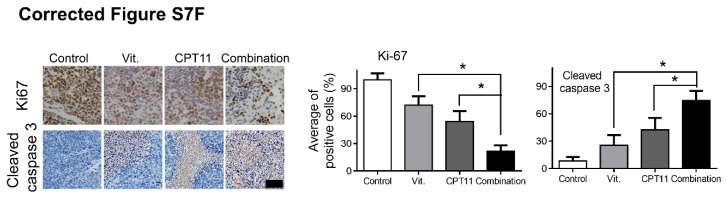
**Corrected Figure S7F** Paraffin-embedded tumor sections were stained with anti-Ki67 or cleaved caspase 3 antibody (scale bar: 50μm), the proliferation and apoptosis index was quantified.

